# Influence of the local structure in phase-change materials on their dielectric permittivity

**DOI:** 10.1186/s11671-015-0735-4

**Published:** 2015-02-05

**Authors:** Kostiantyn V Shportko, Eugen F Venger

**Affiliations:** I. Institute of Physics (IA), RWTH University of Technology Aachen, Sommerfeldstr. 14, Aachen, 52056 Germany; Department of semiconductor heterostructures, V.E. Lashkarev Institute for semiconductor physics of NAS of Ukraine, 45 Nauki av, Kyiv, 03028 Ukraine

**Keywords:** Phase-change materials, Phonon, Dielectric function, 70.78.40.F, 70.63.20.K

## Abstract

Ge-Sb-Te alloys, which belong to the phase-change materials, are promising materials for data storage and display and data visualization applications due to their unique properties. This includes a remarkable difference of their electrical and optical properties in the amorphous and crystalline state. Pronounced change of optical properties for Ge-Sb-Te alloys is linked to the different bonding types and different atomic arrangements in amorphous and crystalline states. The dielectric function of phase-change materials has been investigated in the far infrared (FIR) range. Phonons have been detected by FTIR spectroscopy. Difference of the dispersion of the dielectric permittivity of amorphous and crystalline samples is caused by different structures in different states which contribute to the dielectric permittivity.

## Background

Ge-Sb-Te alloys are characterized by a profound change of optical and electrical properties between the amorphous and crystalline state, which makes them attractive for data storage [1,2] and display and data visualization [3] applications. The electrical resistivity of the amorphous and crystalline phases commonly differs by about 3 to 4 orders of magnitude, which is very favorable for phase-change memory operation [4].

These properties might be used in memories based on PC materials (PCRAM), which operation relies on the fact that chalcogenide-based materials, such as phase-change alloys that contain Ge, Sb, and Te, can be reversibly switched from an amorphous phase (‘0’ state) to a crystalline phase (‘1’ state) or vice versa by an external electric current. PCRAM are a candidate to replace flash memory for non-volatile data storage applications due to their lower power consumption and higher operation speed [5-7].

Phase-change materials have already been employed in optical data storages (CDs, DVDs, Blu-Ray disks) due to the profound change in the optical properties between the amorphous and the crystalline state. In [3], it was demonstrated that by combining the optical and electronic properties of phase-change materials, display and data visualization applications that go beyond data storage applications can be created. This motivates researchers to investigate and to understand the optical properties of the phase-change materials in a wide spectral range.

## Methods

Samples investigated in this study were prepared in a following way: firstly, a 150-nm Al layer was deposited onto a glass substrate. After that, the Ge-Sb-Te film (1,000 nm) was d.c. sputter deposited onto it. In this work, we have investigated two phase-change alloys: Ge_2_Sb_2_Te_5_ and Ge_3_Sb_2_Te_6_. To obtain polycrystalline samples, the as-deposited amorphous films were annealed in Ar atmosphere for 30 min at temperatures about 20°C above their corresponding crystallization temperatures. The structure of the phase-change samples was checked by X-ray diffraction [8]. The phase-change layer thickness was determined on reference samples prepared in the same sputter session using an Alpha-Step 200 profilometer (Tencor Instruments, Milpitas, CA, USA).

IR reflectance spectra were measured at room temperature in the range from 50 to 650 cm^−1^, using a Bruker IFS 66v/s spectrometer with a Hg lamp (Bruker Optics, Ettlingen, Germany) as the radiation source employing a resolution of 1 cm^−1^. Two hundred fifty-six scans were collected in each experiment. A gold mirror (300 nm thick layer of gold, deposited on glass) with a reflectance of 0.99 in the IR served as a reference for the measurements. To normalize the data, the IR reflectance spectra of the reference and the sample were measured subsequently. After dividing the sample’s spectrum by the reference, the final spectrum was obtained. The relative measurement error for the reflectance is 0.2% in the measured wavelength range.

Experimental reflectance spectra were analyzed using the SCOUT software. The model of the dielectric function *ε*(*ν*) used is composed of the following parts: a constant, which is responsible for the polarizability in the higher energy range, a Drude contribution for free charge carriers (in the case of the crystalline samples), and a sum of Kim oscillators to describe the contribution of the IR-active phonons [9,10].

## Results and discussion

**Figure 1:**
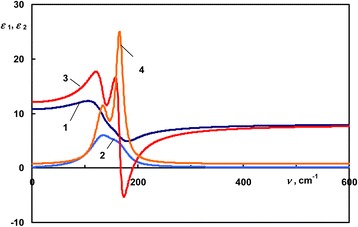
**The dispersion of the dielectric permittivity of AgInTe**_**2**_ **.** 1, 2 - *ε*_1_ and *ε*_2_ of the amorphous sample; 3, 4 - *ε*_1_ and *ε*_2_ of the crystalline sample.

In the non-phase change covalently bonded semiconductor materials [11,12], the short-range order in the amorphous state is essentially the same as in the crystalline state. This we can illustrate on the example of non-phase-change material AgInTe_2_. Figure 1 presents the dispersion of the dielectric permittivity of AgInTe_2_ in the reststrahlen. Curves 1 and 2 correspond to the real and imaginary parts of the dielectric function of the amorphous AgInTe_2_, and curves 3 and 4 are the real and imaginary parts of the dielectric function of the crystalline AgInTe_2_. Both curves 2 and 4 show two phonon peaks at around 136 and 166 cm^−1^. Peaks of curve 2 are lower and wider, since the amorphous state lacks a long-range order of crystalline state. The peaks are placed almost at the same wavenumbers as those in curve 4, because the amorphous state has the same local arrangement as crystal. Short-range order is controlled by a chemical bond, and for AgInTe_2_, it does not change upon transition between amorphous and crystalline state; therefore, *ε*_1amorphous_ ≈ *ε*_1crystalline_ (above 400 cm^−1^).

The amorphous state of phase-change materials is bonded covalently, while the crystalline phase utilizes resonant bonding. Due to this fact, the dielectric permittivity of the crystalline phase above the reststrahlen and below the optical band gap is several times bigger than that of the amorphous phase [13]. Vibrational properties of phase-change materials have been studied using Raman spectroscopy in [14-16]. In this study, we have focused on the dispersion of dielectric function of phase-change materials in the reststrahlen. The dielectric function of two Ge-Sb-Te alloys: Ge_2_Sb_2_Te_5_ and Ge_3_Sb_2_Te_6_, has been investigated.

**Figure 2:**
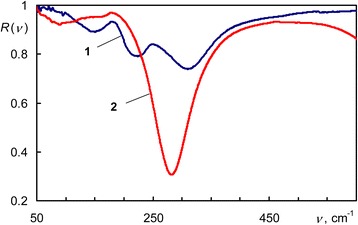
**Experimental reflectance spectra of Ge**_**2**_ **Sb**_**2**_ **Te**_**5**_ **.** 1 - amorphous, 2 - crystalline.

Optical contrast in the IR reflectance spectra of Ge-Sb-Te alloys has been observed in the far infrared (FIR). Experimental reflectance spectra of Ge_2_Sb_2_Te_5_ are shown in Figure 2. Two deep minima above 250 cm^−1^ in curves 1 and 2 are caused by the interference of the IR light in the Ge_2_Sb_2_Te_5_ film, other minima correspond to the phonons’ absorption.

**Figure 3:**
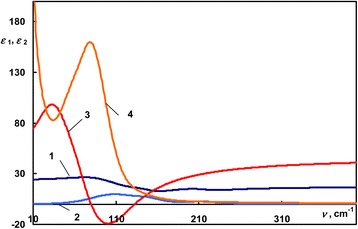
**The dispersion of the dielectric permittivity of Ge**_**2**_ **Sb**_**2**_ **Te**_**5**_ **.** 1, 2 - *ε*_1_ and *ε*_2_ of the amorphous sample; 3, 4 - *ε*_1_ and *ε*_2_ of the crystalline sample.

**Figure 4:**
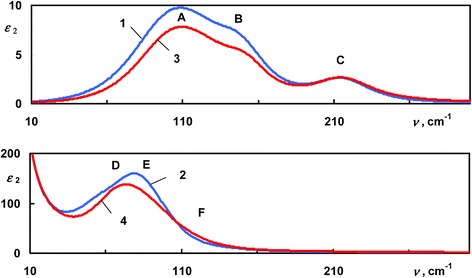
**The dispersion of the imaginary part of the dielectric permittivity of Ge-Sb-Te alloys.** Ge2Sb2Te5: 1 - amorphous, 2 - crystalline; Ge3Sb2Te6: 3 - amorphous, 4 - crystalline.

The dispersion of the dielectric function of amorphous and crystalline Ge_2_Sb_2_Te_5_ was obtained from the fit of IR reflectance spectra, and it is shown in the Figure 3. Curves 1 and 2 correspond to the real and imaginary parts of the dielectric function of the amorphous Ge_2_Sb_2_Te_5_, and curves 3 and 4 are the real and imaginary parts of the dielectric function of the crystalline Ge_2_Sb_2_Te_5_. Unlike non-phase-change AgInTe_2_, one can notice the profound difference between the dielectric function of amorphous and crystalline phase-change Ge_2_Sb_2_Te_5_. On one hand, *ε*_2amorphous_(*ν*) has following peaks at around 115 cm^−1^, ‘shoulder’ 149 cm^−1^, and small peak at 217 cm^−1^. On other hand, *ε*_2crystalline_(*ν*) shows rise at very low wavenumbers; this corresponds to the contribution of free charge carriers and one strong phonon peak near 80 cm^−1^ with a wide slope above 100 cm^−1^. Due to the high electronic polarizability in the crystalline state of Ge_2_Sb_2_Te_5_, *ε*_1crystalline_ > *ε*_1amorphous_ in the range above 250 cm^−1^. The dispersion of the dielectric function of Ge_3_Sb_2_Te_6_ has almost the same appearance in the FIR. Real and imaginary parts of the dielectric function of both alloys have been obtained from the fit of experimental reflectance spectra and are shown in Figure 4. The dielectric function of amorphous Ge-Sb-Te alloys in the FIR has been composed of three oscillators; in the case of crystalline Ge-Sb-Te alloys, 3 oscillators and Drude component were used. Corresponding positions of the phonons’ absorption peaks (in cm^−1^) in the *ε*_2_(*ν*) of studied Ge-Sb-Te alloys are shown in Table 1.

**Table 1:** **Positions of the phonons’ absorption peaks (in cm** ^−1^ **) in the** ***ε***_2_ **(** ***ν*** **) of studied Ge-Sb-Te alloys**

**Peak**	**Ge**_**2**_ **Sb**_**2**_ **Te**_**5**_	**Ge**_**3**_ **Sb**_**2**_ **Te**_**6**_
Amorphous		
*A*	115	116
*B*	149	150
*C*	217	216
Crystalline		
*D*	69	74
*E*	89	91
*F*	135	133

In Ge-Sb-Te alloys, the Ge and Te atoms build the octahedral and tetrahedral structures. A. Kolobov et al. [17] claim that the Ge atoms occupy the octahedral and tetrahedral symmetry positions in the crystalline and amorphous states in Ge-Sb-Te alloys, respectively. Authors [18] have reported the phonon energies in Ge-Sb-Te alloys and assigned them to the vibrations of the structures built from the Ge and Te atoms.

To explain the contrast in the dispersion of the dielectric function between amorphous and crystalline Ge-Sb-Te samples, we compared our experimental results with data from [18]. According to [18], we assigned oscillators used to model the dielectric function of Ge_2_Sb_2_Te_5_ and Ge_3_Sb_2_Te_6_ (Table 1) to the vibrations of Ge-Te octahedra and tetrahedra. In amorphous Ge-Sb-Te, peaks *A* and *B* are close to the energies of the vibrations of Ge-Te octahedra; the peak *C* corresponds to the vibrations of Ge-Te tetrahedra. In case of crystalline Ge-Sb-Te alloys, peaks *D*, *E*, and *F* correspond to the vibrations of Ge-Te octahedra.

## Conclusions

The contrast of the dielectric function of amorphous and crystalline phase-change materials has been observed in the FIR. In this range, the dielectric function is mainly governed by the phonons, and it was modeled by the sum of oscillators, which were attributed to the vibrations of Ge-Te octahedra and tetrahedra. Observed contrast of optical properties of phase-change materials in the FIR is caused by different contributions of the local structures into the dielectric permittivity of Ge-Sb-Te alloys.
